# The influence of the Korean traditional Chungkookjang on variables of metabolic syndrome in overweight/obese subjects: study protocol

**DOI:** 10.1186/1472-6882-13-297

**Published:** 2013-10-31

**Authors:** Hyang-Im Back, Ki-Chan Ha, Hye-Mi Kim, Min-Gul Kim, Ok-Kyeong Yu, Moon-Sun Byun, Do-Youn Jeong, Seong-Yeop Jeong, Youn-Soo Cha, Tae-Sun Park

**Affiliations:** 1 Healthcare Claims & Management Incorporation, 758 Baekie-daero, Deokin-gu, Jeonju, Jeonbuk 561-832, Republic of Korea; 2 Department of Medical Nutrition Therapy, Chonbuk National University Medical School, 567 Baekje-daero, Deokjin-gu, Jeonju, Jeonbuk 561-756, Republic of Korea; 3 Clinical Trial Center, Chonbuk National University Hospital, 20 Geonji-ro, Deokjin-gu, Jeonju, Jeonbuk 561-712, Republic of Korea; 4 Department of Food Science and Human Nutrition, Chonbuk National University, 567 Baekje-daero, Deokjin-gu, Jeonju, Jeonbuk 561-756, Republic of Korea; 5 Obesity Research Center, Chonbuk National University, 567 Baekje-daero, Deokjin-gu, Jeonju, Jeonbuk 561-756, Republic of Korea; 6 Sunchang Research Center for Fermentation Microbes, 61-27 Minsongmaeul-gil, Sunchang-eup, Sunchang, Jeonbuk 595-804, Republic of Korea; 7 Department of Endocrinology and Metabolism, Chonuk National University, Medical School, 20 Geonji-ro, Deokjin-gu, Jeonju, Jeonbuk 561-712, Republic of Korea

**Keywords:** Chungkookjang, Metabolic syndrome, Overweight, Obesity, Diabetes

## Abstract

**Background:**

Metabolic syndrome is a set of disorders that increases the risk of developing cardiovascular disease. The primary target of treatment of patients with metabolic syndrome is therapeutic lifestyle change. Numerous preclinical study have reported positive effects of chungkookjang in *in vivo* models of diabetes and obesity, but there is a paucity of controlled clinical trials on variables of metabolic syndrome in obese subjects. Thus, the objective of this trial is to examine the effect of chungkookjang compared to placebo on variables of metabolic syndrome in overweight/obese subjects.

**Methods:**

This double-blind randomized controlled crossover trial will be conducted on 120 overweight/obese subjects; aged 19–29 years. Subjects will be recruited from the Chonbuk National University, Jeonju, South Korea. Enrolled subjects will be randomly assigned to two groups of equal number; one group received 35 g of chungkookjang (n = 60) and the other group received placebo (n = 60) on a regular daily basis for 12 weeks. After a 12-week washout period, the groups will be crossed over. In addition to anthropometric measures and blood pressure, glucose parameter, lipid profile, adipocytokine, and carnitine assay will be determined at baseline and 12 week. Also, safety will be assessing by measuring total bilirubin, alkaline phosphatase, alanine transaminase, aspartate aminotransferase, total protein, albumin, blood urea nitrogen, creatinine, and creatine kinase at baseline and 12 weeks. 24-hour dietary recalls were collected at the baseline and at the end of the trial.

**Discussion:**

This trial will evaluate the effects of chungkookjang on variables of metabolic syndrome in overweight/obese subjects. The results of this study may contribute to the reduction of risk factor for metabolic syndrome caused by obesity.

**Trial registration:**

Clinical trials NCT01811511.

## Background

The prevalence of obesity has increased worldwide over the past decades [1,2], although more recent data suggest a slowing of this trend. The rising rates of obesity in children and adults have been accompanied by an increase in the co-occurrence of obesity-associated metabolic abnormalities known as the metabolic syndrome (Met-S). The prevalence of Met-S is increasing; it currently affects 22.1% of men and 27.8% of women in Korea [3,4]. The clustering of major components of the syndrome, including obesity, dyslipidemia, hypertension, and insulin resistance, has been demonstrated in youth [5,6], and clustering of these risk factors has also been shown to track into early adulthood. These obese youth are a high-risk population to target for screening, prevention, and intervention for Met-S [5,6].

Conclusions in nutritional intervention studies are frequently drawn from epidemiological studies, but there are affected by many possible confounding factors. Unlike laboratory animals, human beings cannot be fed over a long time; thus most nutritional intervention studies are short to medium term and focus on surrogate parameters. On the other hand, although the preferred treatment for Met-S is lifestyle modification including exercise and nutrition therapy [7,8], the challenge is the multitude of risk factors and the high prevalence of obesity. But there are difficult to approach in typically clinic visits. Furthermore, in the large scale trials, subjects often struggle with adopting lifestyle changes to derive siginificant results, but it is difficult to control the subjects by physician or investigators.

Soybeans contain various nutritious and functional components such as isoflavonoids besides soy protein which are helpful in protecting against metabolic diseases such as obesity and type 2 diabetes [9]. Fermenting soybean such as chungkookjang (CKJ) may be enhance the functionality of soybeans to protect against metabolic disease. Korean traditional CKJ is a good fermented soybean product for use as a functional food. However, traditional CKJ did not recognized as a functional food in Korea. Currently, the non-clinical data on effects of CKJ have been reported from in vivo and in vitro studies [10-18] but there is paucity the controlled clinical trials on variables of Met-S. Our previous study have revealed that traditionally made CKJ from Sunchang district of Jeolla province, Korea showed potential anti-atherosclerotic effects in overweight/obese subjects [19]. It has also been reported that traditional CKJ (made from Sunchang district of Jeolla province, Korea) improves insulinotropic action and hepatic insulin sensitivity in diabetic rats [16,17].

However, CKJ intake in Korean youth is gradually decreased due to distinctive flavor of CKJ. Therefore, the present study will be conducted by the easy-to-eat freeze-dried CKJ pills. The CKJ shows distinctive flavor characteristics. For this reason, we used cinnamon as flavoring agent to make it palatable. The purpose of this study is to evaluate the effect of CKJ compared to placebo on variables of Met-S in overweight/obese subjects.

## Methods and design

### Objective

The objectives of this RCT are to study whether the CKJ can improve the Met-S in overweight/obese subjects.

#### Primary objective

To evaluate the efficacy of the CKJ on anthropometric parameter in overweight/obese subjects after 12 weeks of consumption.

#### Secondary objectives

To evaluate the following factors in overweight or obese subjects after 12 weeks of consumption on:

A) blood pressure

B) glucose parameter

C) lipid profile

D) adipocytokine

E) carnitine assay

### Design and setting

**Figure 1:**
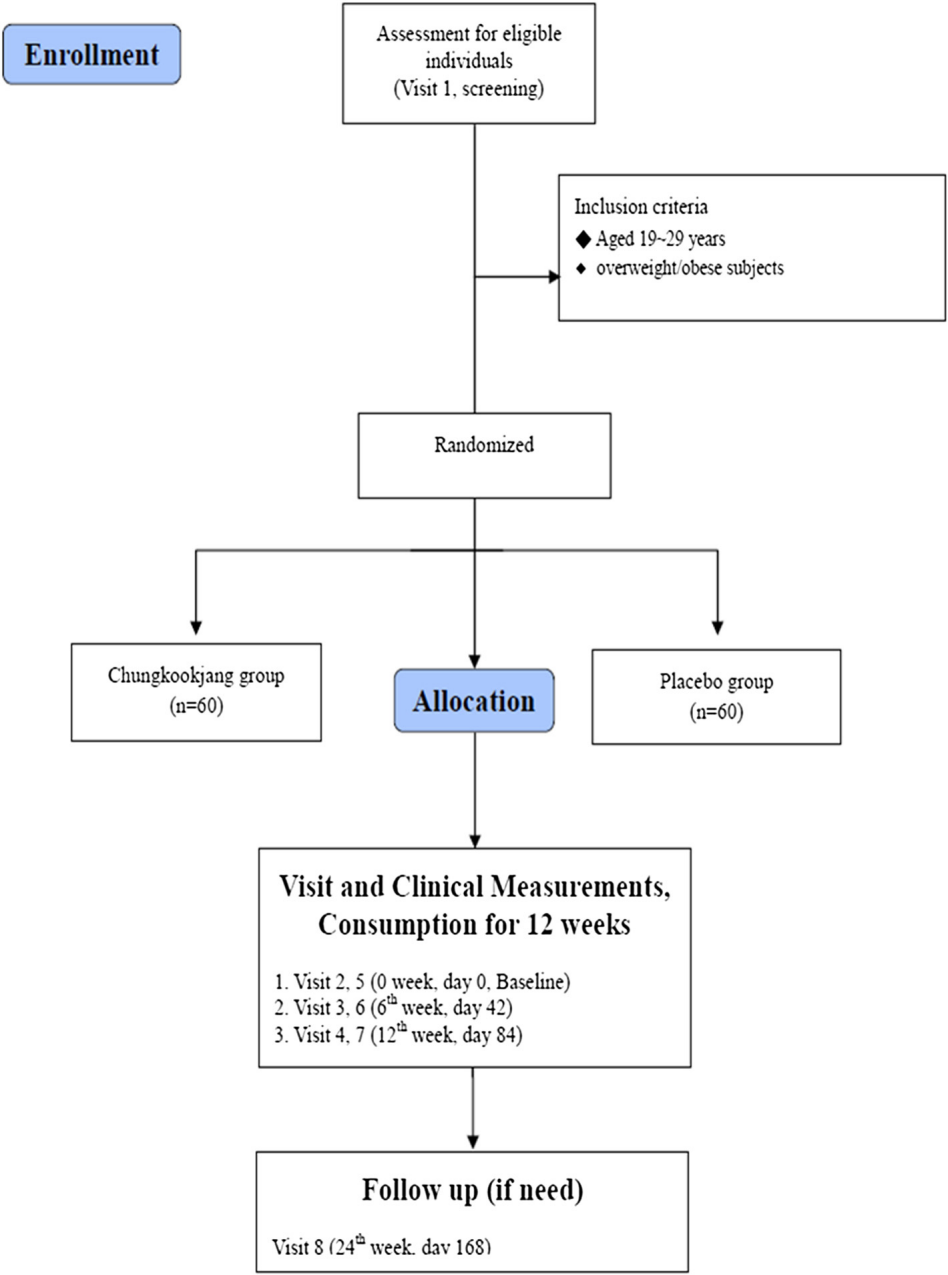
**Flow diagram for study.** Summary of the study flow.

This is a 12 weeks, randomized, double-blinded, placebo-controlled crossover study. From February 2013 to December 2013, Obesity Research Center of Chonbuk National University and Chonbuk National University Hospital Clinical Trial Center in Korea will participate in this study. Recruitment will therefore be expected to be completed within the study period. To recruit the participants, we will advertise in the bulletin board and on the internet homepage of Chonbuk National University. Eligible participants will be randomly allocated into 2 group (the CKJ or placebo group) with 1:1 allocation ratio and receive treatment for 12 weeks. After a 12-week washout period, the groups will be crossed over. The evaluation of participants and the analysis of the results will be performed by professional blinded to the group allocation. Written consent will be obtained from each participant before the start of this study. Our study plan is summarized in Figure 1.

### Inclusion criteria

Subjects will be included if they meet the following criteria: (1) men and women between 19 and 29 years of age; (2) overweight/obese (body mass index of 23 kg/m2 or a waist circumference of 90 cm for men, 80 cm for women); (3) ability to give informed consent.

### Exclusion criteria

The exclusion criteria for the study were: (1) severe heart disease (heart failure, angina, myocardial infarction, valvular heart disease, arrhythmias); (2) severe cerebrovascular disease (stroke, hemorrhage); (3) acute or chronic inflammatory disease; (4) malignant tumor (leukemia, lymphoma); (5) diagnosis of type 1 and type 2 diabetes or fasting glucose ≥ 126 mg/Dl; (6) Systolic pressure ≥ 160 mmHg, diastolic pressure ≥ 100 mmHg; (7) hereditary hyperlipidemia; (8) allergic or hypersensitive response to any of the ingredients in the test products; (9) a history of disease that could interfere with the test products or impede their absorption such as gastrointestinal disease or gastrointestinal surgery; (10) under antipsychotic drugs therapy within past 2 months; (11) participation in any other clinical trials within past 2 months; (12) abnormal hepatic liver function or renal disease (acute/chronic renal failure or nephrotic syndrome); (13) laboratory test, medical or psychological conditions that might interfere with successful participation in the study based on the judgment of the investigators.

### Randomization and allocation concealment

After enrollment, subjects will be randomly assigned to one of the two groups, either the CKJ group or placebo group. The allocation ratio will be 1:1 in blocks of 2. Randomization will be performed at a site remote from trial location. Random numbers will be generated by a computerized random-number generator through the block-randomization method of a software program (Excel, Microsoft Office 2007) for sequence generation. At the time of randomization subjects will draw an envelope. Each envelope contains a number that is concealed to the treatment allocation. Randomization sequence and allocation will be concealed to all study subjects, research staff, investigators and pharmacists until completion of the study. The allocation list will be protected by password access files and held by a non-investigator independent. In the event of an emergency medical situation the individual’s randomization code and group allocation can be identify.

### Intervention

Subjects will be assigned CKJ or placebo group for 12 weeks. CKJ treatment group will be received 35 g CKJ (equivalent to 70 g of fresh CKJ) per day and they should consume a pack of the CKJ with three times a day after meals. The selection of the doses investigated in this study was based upon efficacy and safety data obtained from a previous human study [19]. Chungkookjang is provided by Sunchang Research Center for Fermentation Microbes (Sunchang, Republic of Korea), freeze-dried using a freeze-dryer, and then made into pills (Imshil Herbal Medicine Co., Imshil, Republic of Korea). Placebos were made with the same taste, appearance, and energy content but without the principal ingredient that was present in the CKJ. CKJ/placebo pills were provided to subjects every 6 weeks.

### Outcome measures

#### Primary outcome measurement

The primary outcome is the anthropometric parameter (weight, body mass index, body fat mass, percentage body fat, lean body mass, waist circumference, hip circumference, and waist to hip ratio).

#### Secondary outcome measurements

Secondary outcomes will be blood pressure, glucose parameter (fasting glucose, HbA1c, insulin, gamma-glutamyl transferase [GGT]), lipid profile (total cholesterol, triglycerides, high-density lipoprotein-cholesterol [HDL-C], low-density lipoprotein-cholesterol [LDL-C], free fatty acid, apolipoprotein A1 [Apo A1], apolipoprotein B [Apo B], high-sensitivity C-reactive protein [hs-CRP]), adipocytokine (leptin, adiponectin, ghrelin), and carnitine assay. These biomarkers will be checked at weeks 0 (baseline) and 12 (end of the trial). 24-hour dietary recalls were collected at the baseline and at the end of the trial. The schedule of assessments is presented in Table 1.

**Table 1:** A brief study schedule at every visit

	***Crossover design***				
	***Screening***	***Baseline***			***Follow up***
	***Visit 1***	***Visit 2, 5***	***Visit 3, 6***	***Visit 4, 7***	***(Visit 8)***
	***D-28***	***Week 0***	***Week 6***	***Week 12***	***Week 24***
	***~D-1***	***D0***	***D42***	***D84***	***D168***
***Informed consent form***	◯				
***Demographic information taking***^***1***^	◯				
***Medical history taking***	◯				
***Inclusion/exclusion criteria check***	◯	◯			
***Physician examination***^***2***^		◯		◯	◯
***Drinking/smoking taking status***^***3***^		◯		◯	
***Vital sign measurement***	◯	◯	◯	◯	◯
***Concomitant drugs check***	◯	◯	◯	◯	◯
***Anthropometric measures***^***4***^		◯	◯	◯	◯
***Glucose parameter***^***5***^		◯		◯	
***Lipid profile***^***6***^		◯		◯	
***Adipocytokine***^***7***^		◯		◯	
***Carnitine assay***		◯		◯	
***Laboratory test***^***8***^		◯		◯	
***Study product distribution***		◯	◯		
***Compliance checking***			◯	◯	
***Adverse event monitoring***		◯	◯	◯	◯
***Diet, physical exercisecounseling***^***9***^		◯	◯	◯	◯

◯: Item need to be performed during the visit.

^1^ Sex, date of birth, age, contact address and phone number.

^2^ Include present illness, past history taking.

^3,9^Risk factors such as alcohol drinking, smoking, severe exercise and other dietary supplement intake will be managed.

^4^ weight, body mass index, body fat mass, percentage body fat, lean body mass, waist circumference, hip circumference, and waist to hip ratio.

^5^ fasting glucose, HbA1c, insulin, gamma-glutamyl transferase (GGT).

^6^ total cholesterol, triglycerides, high-density lipoprotein-cholesterol (HDL-C), low-density lipoprotein-cholesterol (LDL-C), free fatty acid, apolipoprotein A1 (Apo A1), apolipoprotein B (Apo B), high-sensitivity C-reactive protein (hs-CRP).

^7^ leptin, adiponectin, ghrelin.

^8^ Blood test (white blood cell [WBC], red blood cell [RBC], hemoglobin, hematocrit, platelets count, total bilirubin [TB], alkaline phosphatase [ALP], alanine transaminase [ALT], aspartate aminotransferase [AST], total protein, albumin, blood urea nitrogen [BUN], creatinine, creatine kinase [CK]), urine test (specific gravity, pH, nitrite, protein, glucose, ketone, urobilinogen, bilirubin, microscopic RBC/WBC).

### Statistical analysis and sample size

#### Baseline data and outcomes data

Statistical analysis will be performed using SAS version 9.3 for Windows (SAS Institute, Cary, NC, USA). Data will be presented as mean values and standard deviation (SD). The Chi-square test will be performed to determine differences at baseline in frequencies of categorized variables between the groups. A linear mixed-effects model will be applied to repeated-measures data for each continuous outcome variable. Fixed effects will be included treatment group, treatment visit, and interaction between treatment group and visit. When the analysis of variance indicated significant differences among groups, post hoc test (Turkey’s test) will be used to separate the differences between groups before and after the 12-week intervention period. A value of p < 0.05 will be considered statistically significant.

#### Compliance

The CKJ or placebo remaining after each visit will be quantified in order to enhance medication compliance. Subjects whose compliance with the CKJ or placebo is ≤ 70% of the total dose will be considered to have dropped-out.

#### Sample size

Sample size calculation is performed; primary outcome measure will be the percentage body fat, whereby 1.1% is assumed a clinically relevant difference between the two groups, with a standard deviation of 2.78%, alpha set on 5% and power on 80%. This resulted in a required number of 50 subjects in each group. Assuming a dropout rate of 20%, a total of 120 subjects will be enrolled in this trial.

### Adverse events

All unexpected adverse events related to CKJ intake will be reported to the investigator by subjects and write on the individual case report form by the investigator. Safety will be assessed by the reporting of clinical laboratory tests, vital sign measurements, and adverse events. Clinical laboratory tests, including blood test (white blood cell [WBC], red blood cell [RBC], hemoglobin, hematocrit, platelets count, total bilirubin [TB], alkaline phosphatase [ALP], alanine transaminase [ALT], aspartate aminotransferase [AST], total protein, albumin, blood urea nitrogen [BUN], creatinine, creatine kinase [CK]), and urine test (specific gravity, pH, nitrite, protein, glucose, ketone, urobilinogen, bilirubin, microscopic RBC/WBC) will be determined at weeks 0 (baseline) and 12 (end of the trial). Vital signs of each subject will be checked with monitoring of AEs (any AEs related to CKJ intake) after each visit.

### Participant protection and ethics

The study protocol and the written informed consent were approved by the Functional Foods Institutional Review Board of Chonbuk National University Hospital (CUH IRB 2013-01-002). Each subject will be notified regarding the study protocol. Written informed consent will be obtained from each subject.

## Discussion

The aim of this study is to evaluate the efficacy of the CKJ on the Met-S. For this purpose, the ability of the CKJ to variables the Met-S will be assessed in 120 overweight/obese subjects.

In this study, we expect that the CKJ effectively reduction of risk factor for Met-S. Some studies have investigated the action of CKJ on the Met-S. The clinical trial for Met-S enhancement of CKJ has not been performed up to this time. However, numerous studies have reported from in vivo and in vitro studies [10-19]. Therefore, if this study will be successfully performed, the CKJ may offer beneficial effects in reducing of risk factor for Met-S. And these results will have important implications for the design of sustainable cost-effective health services for people with Met-S caused by obesity. In addition, this study will be the groundwork for the larger scale RCT. To draw confirmative conclusion about the therapeutic efficacy and safety of the CKJ, a full-scale RCT will be performed.

## Competing interests

The authors declare that they have no competing interests.

## Authors’ contributions

KCH, HIB, HMK, DYJ, SYJ, YSC, and TSP received the research funding, led the entire study, and drafted the manuscript. MGK, OKY, and MSB participated in the design of the study and performed the statistical analysis. All authors read and approved the final manuscript.

## Pre-publication history

The pre-publication history for this paper can be accessed here:

http://www.biomedcentral.com/1472-6882/13/297/prepub
